# Enabling patient–physician continuity in Swedish primary care: the importance of a named GP. A registry-based observational study

**DOI:** 10.3399/BJGPO.2024.0118

**Published:** 2024-10-16

**Authors:** Lina Maria Ellegård, Anders Anell, Gustav Kjellsson

**Affiliations:** 1 Department of Economics, Lund University, Lund, Sweden; 2 Kristianstad University, Kristianstad, Sweden; 3 Department of Business Administration, Lund University, Lund, Sweden; 4 Centre for Health Governance, Department of Economics, School of Public Health and Community Medicine, Gothenburg University, Gothenburg, Sweden

**Keywords:** Continuity of care, Practice management, Chronic conditions

## Abstract

**Background:**

Continuity of care is important for patients with chronic conditions. Assigning patients to a named GP may increase continuity.

**Aim:**

To examine whether patients who were registered with a named GP at the onset of their first chronic disease had higher continuity of care at subsequent visits than patients who were only registered at a practice.

**Design & setting:**

Registry-based observational study in Skåne County, Sweden. The study population included 66 063 patients registered at the same practice at least 1 year before the onset of their first chronic condition between 2009 and 2015.

**Method:**

We compared patients registered with a named GP with patients only registered at a practice over a 4-year follow-up period. The primary outcome was the usual provider of care (UPC) index for all visits and for visits related to the chronic disease. Secondary outcomes were the number of GP, nurse, and out-of-hours visits; emergency department visits; hospital admissions; and mortality. We used linear regression models, adjusted for patient characteristics (using entropy balancing weights) and for practice-level fixed effects, to compare the UPC between those registered with a named GP and those who were not.

**Results:**

Patients with a named GP at onset of their condition had a UPC that was 3–4 percentage points higher than patients who did not have a named GP, but the difference decreased and was not statistically significant after adjusting for patient and practice characteristics. Patients with a named GP made more visits, although not specifically for the chronic condition. There were no statistically significant differences for the other outcomes.

**Conclusion:**

Patient registration with a GP at diagnosis of their first chronic condition does not demonstrate higher continuity of care at subsequent GP visits and is not linked to other relevant outcomes for patients.

## How this fits in

Continuity of care in group practices may be higher if patients are registered with a named GP. This observational study examined continuity of care for patients after the onset of their first chronic condition. Patients who were registered at a named GP did not have higher continuity of care than patients without a named GP. Registering patients with a named GP in a group practice is not guaranteed to improve continuity of care.

## Introduction

Continuity is a central component of primary care.^1^ A continuous relationship may improve outcomes by increasing GPs’ personal responsibility for and knowledge about patients, and may also support the development of mutual trust.^2^ Empirical evidence links continuity to increased efficiency,^3^ reduced hospitalisations, and lower mortality.^4–10^ Continuity of care may be particularly important for patients with higher levels of need, such as older individuals or those with chronic conditions, and is greatly valued by such patients.^11,12^

In a number of countries, primary care has become more fragmented as a result of its development from the traditional solo practice organisation towards larger, multiprofessional group practices with an increasing number of GPs working part-time and sharing patient lists.^9,13^ Fostering continuity in modern-day primary care requires new ways of working, both at the GP and practice level,^14^ but research to inform this transition is limited.^9^ Registering patients with a named GP has been suggested as a way to build continuity.^15^ A previous study found that a mandate in the English NHS to allocate patients a named GP did not increase continuity of care.^16^ However, effects on important subgroups such as patients with a chronic condition may have been masked as a result of the short follow-up period.

The present study explores the importance of having a named GP for developing continuity in Swedish multiprofessional group practices, primary care centres (PCC). Patients have the right to be registered with a PCC, but registration with a named GP is far from guaranteed.^17^ The aim of the study was to examine whether patients with a named GP had higher relational continuity following the onset of their first chronic condition compared to patients with no named GP.

## Method

### Setting

The regional healthcare system in Skåne County (1.4 million residents) contains around 150 PCCs, around half of which are privately owned. All PCCs employ GPs, nurses, and physiotherapists; some also employ behavioural therapists.^17^ PCCs are mainly reimbursed by capitation, adjusted for morbidity (using the Johns Hopkins adjusted clinical groups [ACG] system) and sociodemographic factors.

All residents are registered at a PCC. The default PCC is the closest one, but patients may freely choose where to register (PCCs cannot close their lists). PCCs may decide whether to register patients with a specific (named) GP. Patients can seek care at any PCC, but normally visit the one they are registered with.

### Data

The regional care registers record all contacts (date, diagnoses, type of professional, practice or clinic identifier) with publicly funded healthcare and registrations with PCCs (start date, end date, PCC identifier, named GP [if any]). The data were linked to patient background data from registers held by Statistics Sweden using a personal identifier. The dataset spanned 2005–2019 (data for 2005–2008 were used to identify any previous diagnoses).

### Study population, follow-up, and exposure

To focus on chronic conditions that are normally managed by Swedish PCCs, the authors considered the following set of conditions: Alzheimer’s disease, asthma, type 2 diabetes, depression, heart failure, hypertension, ischaemic heart disease (IHD), chronic obstructive pulmonary disease, or anxiety. The study population included 66 063 patients who, i) in 2009–2015, had their first record of any of the above diagnoses (counting from 1 Jan 2005); ii) had no record of any other chronic condition during the 4 years before the date of the first diagnosis (index date; other chronic conditions were defined using the chronic condition indicator in the ACG system); and iii) had been registered at the same PCC (with the same GP identifier) for at least 365 days before the index date. The supplementary material (section 2) gives details of the International Classification of Diseases (ICD) codes and the construction of the sample.

Outcomes were studied over a follow-up period of 4 years after the index date. Patients who died or moved out of the region were not excluded from the analysis, but the effective timeframe used to compute outcomes for them was thus shorter than 4 years (Table 1).

**Table 1.** Follow-up and censoring

	Patient 1	Patient 2
Index date (DD/MM/YYYY)	01/02/2013	01/02/2013
Last date of follow-up	31/01/2017	31/01/2017
Censored	No	Yes
Censoring date	N/A	31/01/2015
Effective follow-up	4 years	2 years

The table illustrates the outcomes computed for two fictional patients with the same index date, one of whom was censored (because they died) during the follow-up period. Outcomes (number of visits, continuity of care) are counted over the full follow-up period for both patients, but the effective follow up is shorter for patient 2 because she is only observed for 2 years after the index date.

Patients were defined as 'exposed' if they were registered with a named GP on the index date, whereas those in the comparison group were only registered at a PCC. The classification as exposed or non-exposed patients did not change, thus patients were considered exposed in the analysis even if they lost their named GP during follow-up.

### Outcomes

The primary outcome was the usual provider of care (UPC) index, which is the share of the patient’s GP visits that were made with the most commonly seen GP (the usual provider [UP]):^18^


$$UPC=\frac{\sum_{t=1}^{4}visits_{t}^{UP}}{\sum_{t=1}^{4}visits_{t}}$$

We computed two versions of the UPC: one for all visits, and one for visits with a registered diagnosis related to the chronic condition; both were computed over the entire follow-up period, and only for patients with at least three GP visits during the period ( $\sum_{t=1}^{4}visits_{t}\geq 3$ ).

Secondary outcomes included the number of GP visits (all diagnoses, and visits with the chronic condition) or nurse visits, and indicator variables for having visited an out-of-hours (OOH) clinic, a hospital emergency department (ED), or being hospitalised (unplanned) at least once. These outcomes were defined over the entire follow-up period. To study attrition, we examined the proportion of patients who were still registered at a PCC in the region, and the proportion of patients who had died, within 1, 2, 3, or 4 years after the index date.

### Background characteristics

We defined variables describing the pre-diagnosis care utilisation (corresponding definitions as for the secondary outcomes) and PCC registrations (period and date of registration, switches). Background characteristics included age (in years), sex, household disposable income (decile), educational attainment (primary, secondary, or tertiary), and indicators for non-Nordic background and recently having moved from another municipality. Time-variant characteristics were measured on 31 December before the index date. See supplementary file section 1 for definitions.

### Statistical analysis

We used least squares regressions to estimate associations between the outcomes and the indicator for being registered with a named GP. Model 1 was unadjusted, and model 2 accounted for observable patient confounders by weighting. To obtain weights, we used entropy balancing (EB) which is a computationally attractive, data-driven alternative to the manual, trial-and-error based preprocessing step in ordinary propensity score modelling.^19^ The EB algorithm derives weights that make the sample moments (such as mean, variance) of the covariates in the comparison group come as close as possible to the moments in the treatment group, while keeping the weights as close as possible to the value 1 to retain information. Balance is thus ensured by construction. We balanced the groups on the first two moments of the variables that i) measured prior primary care utilisation or ii) had modest/large (≥0.05) standardised mean differences (SMD)^20^ (see Supplementary material section 3). Model 3 added PCC fixed effects (FE). By including FEs, the analysis relies only on variation between patients within a given PCC. Thus, the estimated association between the outcome and being registered at a named GP in model 3 does not reflect differences in practice characteristics such as size or location, which may be correlated with the propensity to register patients at a named GP. In all models, standard errors were clustered at the PCC on the index date.

As sensitivity analyses, we included (linear) control variables instead of weighting and included some balanced, but theoretically motivated, covariates (prior ED/OOH visits and hospitalisations) and a covariate that was unbalanced but correlated with the included primary care utilisation variables (the number of GPs seen). We also estimated the UPC model with a shorter follow-up of 2 years.

The analysis was performed using Stata (version 18.1). Entropy balancing weights (EBWs) were obtained using the ebalance package.

## Results

### Descriptive statistics

#### Background characteristics

Table 2 shows descriptive statistics for the background characteristics used to obtain entropy weights. Patients with a named GP were older, had lower educational attainment but higher household income, and were less likely to be non-Nordic immigrants or to have recently moved between municipalities. They registered at a PCC in the region earlier (in chronological time and relative to the index date). A higher proportion had switched PCCs or GPs during the period before the onset of the chronic condition. They were less likely to have depression and more likely to have hypertension or IHD as their first chronic condition (SMD *<*0.05 for the other conditions). The named GP group had higher primary care and ED utilisation 3–4 years before the index date, but their primary care utilisation was lower in the 2 years before the index date. Most differences were small (see Supplementary material section 4 for the SMDs of the full set of background characteristics). The three largest differences were observed for the start of registration date (SMD = -0.776), the index spell duration (SMD = 0.388) and the indicator for having moved (SMD = 0.176).

**Table 2.** Sample moments by group

	Named GP (*n* = 23 762)			No named GP (*n* = 42 301)					
					*A. Before balancing*			*B. After balancing*	
Variable	Mean	Variance	Skewness	Mean	Variance	Skewness	Mean	Variance	Skewness
Age, years	47.4	4422	26	44.2	5468	24.1	47.4	4424	26.1
Education	0.889	0.605	0.194	0.939	0.65	0.111	0.889	0.605	0.194
Disp. inc. (decile)	5.64	8.17	-0.0662	5.36	8.86	0.0283	5.64	8.17	-0.0604
Born in Sweden or Nordic region	0.883	0.103	-2.39	0.849	0.128	-1.94	0.883	0.103	-2.39
Mover^a^	0.0615	0.0577	3.65	0.111	0.0985	2.48	0.0615	0.0577	3.65
Start date (reg.)	17 835	858 335	0.0377	18 432	323 954	0.117	17 835	858 361	-0.07
Duration (reg.)	1298	611 174	1.31	1038	288 864	0.958	1298	611 158	1.4
Registered t-2	0.866	0.116	-2.15	0.817	0.15	-1.64	0.866	0.116	-2.15
Registered t-3	0.72	0.201	-0.983	0.679	0.218	-0.765	0.72	0.201	-0.983
Switched PCC (2–4 years pre)	0.622	0.235	-0.505	0.481	0.25	0.0744	0.622	0.235	-0.504
Switched GP (2–4 years pre)	0.703	0.209	-0.89	0.543	0.248	-0.173	0.703	0.209	-0.89
First diagnosis of depression	0.338	0.224	0.683	0.373	0.234	0.523	0.338	0.224	0.683
First diagnosis of hypertension	0.318	0.217	0.783	0.28	0.202	0.978	0.318	0.217	0.783
First diagnosis of ischaemic heart disease	0.0529	0.0501	3.99	0.0419	0.0402	4.57	0.0529	0.0501	4
Index year	2012	4.42	0.0325	2013	2.59	-0.333	2012	4.42	0.0972
Any GP visit (3–4 years pre)	0.522	0.25	-0.0861	0.495	0.25	0.0201	0.521	0.25	-0.086
Number of GP visits (3–4 years pre)	1.33	3.9	2.87	1.23	3.56	2.68	1.33	3.9	2.91
Number of nurse visits (3–4 years pre)	1.3	11.5	7.82	1.29	10.9	7.05	1.3	11.5	8.91
Any ED visit (3–4 years pre)	0.131	0.114	2.19	0.114	0.101	2.44	0.131	0.114	2.19
Any GP visit (1–2 years pre)	0.867	0.115	-2.16	0.884	0.102	-2.4	0.867	0.115	-2.16
Number of GP visits (1–2 years pre)	2.76	7.21	2.31	2.83	7.11	2.16	2.76	7.21	2.36
Number of nurse visits (1–2 years pre)	2.18	18.2	6.44	2.67	27.2	4.11	2.18	18.2	4.79

The table shows the first three sample moments — the mean, the variance, and the skewness — for the background characteristics which were used to obtain entropy weights. For the comparison group with no named GP, A shows the moments as observed in the data, and B shows the moments after using the entropy weights. The start date and duration variables refer to the PCC registration that was ongoing at the index date. The dummy variables for being registered in t-2 and t-3 refer to the second and third year before the index date (by definition, everyone was registered the year before the index date). The dummy variables for having switched PCC or GP indicate whether such a switch occurred during the 2–4 years before the index date. The prior care use variables at the bottom of the table are calculated over the 3–4 and 1–2 years before the index date, respectively.

^a^An individual who recently moved from another municipality.

ED = emergency department. Disp. inc = disposable income. PCC = primary care centre. Pre = period before the index date. Reg = registration.

After weighting, the means and variances were highly balanced by construction (Part B of Table 2). Notably, the skewness was also balanced for most variables.

#### Outcome variables

The descriptive statistics in Table 3 show that patients with a named GP had slightly higher UPC in the post period (4 years after the index date of the chronic condition), both for all diagnoses (0.484 versus 0.457) and for the chronic condition (0.675 versus 0.638). Only *n* = 17 044/23 762 patients with a named GP made ≥3 GP visits during the follow-up period, whereas *n* = 28 591/42 301 patients without a named GP made ≥3 GP visits during this period; similarly, relatively few had ≥3 visits with a diagnosis related to the first condition (6412 patients with a named GP versus 11 108 without a named GP).

**Table 3.** Summary statistics for main outcome variables by group

	Named GP		No named GP			
	*Obs*	*Mean*	*SD*	*Obs*	*Mean*	*SD*
*Continuity*						
UPC	17 044	0.484	0.236	28 591	0.457	0.217
UPC first	6412	0.675	0.255	11 108	0.638	0.248
*Care utilisation*						
Number of GP visits	23 762	5.95	5.29	42 301	5.32	5.04
Number of GP visits for first chronic condition	23 762	1.83	2.52	42 301	1.73	2.49
Number of nurse visits	23 762	5.18	8.41	42 301	5.01	8.89
Any OOH visit	23 762	0.175	0.38	42 301	0.19	0.392
Any ED visits	23 762	0.354	0.478	42 301	0.348	0.476
Any unplanned hospitalisation	23 762	0.233	0.423	42 301	0.226	0.418
*Attrition*						
Still registered t+1	23 762	0.974	0.16	42 301	0.968	0.177
Still registered t+2	23 762	0.956	0.204	42 301	0.944	0.23
Still registered t+3	23 762	0.94	0.237	42 301	0.924	0.265
Still registered t+4	23 762	0.925	0.263	42 301	0.907	0.291
Death (1 year)	23 762	0.0152	0.122	42 301	0.0134	0.115
Death (2 years)	23 762	0.0241	0.153	42 301	0.0208	0.143
Death (3 years)	23 762	0.0335	0.18	42 301	0.0289	0.167
Death (4 years)	20 703	0.0454	0.208	35 511	0.0396	0.195

The table shows the mean (or proportion for binary variables) and standard deviation (SD) of the outcome variables for the sample used in each model (Obs). The sample generally includes the whole study population, with the following exceptions: 1) For the two continuity variables (usual provider of care [UPC] index), the sample includes individuals with at least three GP visits during follow-up; for visits with any diagnosis (UPC) and for visits related to the new chronic condition (UPC first). 2) For the variable indicating mortality in t+4, the sample excludes patients diagnosed in 2015, because of a lack of data regarding deaths in the final follow-up year for that cohort.

ED = emergency department. OOH = out of hours.

The group with a named GP had slightly higher care utilisation, except that a smaller proportion visited an OOH clinic. More than 90% were still registered at a PCC in the region 4 years after the index date. The proportion was slightly higher in the named GP group, which also had higher mortality. Notably, for the subsample of patients with at least three GP visits in the follow-up period (that is, the subsample used to study the UPC), the overall level of attrition was lower; 95% of patients were uncensored throughout the 4-year follow-up period, 97% were observed for 3 years; 98.5% for 2 years; and 99.5% for at least 1 year.

### Regression estimates

#### Continuity

Table 4 shows the estimates of continuity (UPC). The difference between the groups was only statistically significant in the unadjusted model (1). The difference was reduced by the EBWs (model 2) and, in particular, after accounting for PCC FEs (model 3). In model 3, the magnitude of the difference was close to zero for all visits and around 2 percentage points for visits related to the new chronic condition (for reference, the UPC for the first condition is around 67%, Table 3). In the sensitivity analyses, the UPC estimates were similar; the UPC for the first diagnosis was statistically significant (5% level) when using a control strategy instead of weighing, but not when accounting for the number of GPs seen (Supplementary material section 5).

**Table 4.** Continuity of care

	Any diagnosis			Chronic diagnosis		
	1	2	3	4	5	6
Named GP	0.0276^a^ (0.0139)	0.0264 (0.0169)	-0.000322 (0.00756)	0.0379^a^ (0.0177)	0.0330 (0.0299)	0.0185 (0.0135)
*n*	45 635	45 635	45 635	17 520	17 520	17 520
Entropy balancing weights	No	Yes	Yes	No	Yes	Yes
PCC FE	No	No	Yes	No	No	Yes

The table shows the estimated association (regression coefficients) between having a named GP and continuity of care for three model specifications. Model 1 (columns 1 and 4) shows the unadjusted difference in means. Model 2 (columns 2 and 5) applies entropy balancing weights. Model 3 (columns 3 and 6) applies entropy balancing weights and includes practice fixed effects (PCC FE). Continuity is measured by the usual provider of care (UPC) index for patients with at least three GP visits during follow-up. Standard errors (in brackets) are clustered by PCC.

^a^ *P<*0.05

#### Secondary outcomes

Table 5 shows the estimates for the secondary outcomes relating to care utilisation. Section A shows that patients with a named GP made 0.36 more visits than the comparison group adjusting for patient and PCC characteristics (column 3), but the difference was close to zero and statistically insignificant for the subset of visits with the new chronic diagnosis (column 6). There were no other significant differences after adjusting for patient and PCC characteristics (model 3).

**Table 5.** Secondary outcomes (utilisation)

	1	2	3	4	5	6
A	Number of GP visits (any diagnosis)			Number of GP visits (chronic diagnosis)		
Named GP	0.628^c^	0.411^b^	0.364^c^	0.104	0.0642	-0.0112
	(0.130)	(0.143)	(0.0915)	(0.0590)	(0.0759)	(0.0562)
B	Number of nurse visits			Any OOH visit		
Named GP	0.164	-0.326	0.104	-0.0147	0.00944	0.00605
	(0.198)	(0.271)	(0.183)	(0.0109)	(0.0129)	(0.00599)
C	Any ED visit			Any hospitalisation		
Named GP	0.00630	0.0438^a^	0.0128	0.00708	-0.0106	0.00149
	(0.0143)	(0.0215)	(0.00854)	(0.00555)	(0.0108)	(0.00704)
*n*	66 063	66 063	66 063	66 063	66 063	66 063
EBW	No	Yes	Yes	No	Yes	Yes
PCC FE	No	No	Yes	No	No	Yes

The table shows the estimated associations between having a named GP and the secondary outcomes. Model 1 (columns 1 and 4) shows the unadjusted difference in means. Model 2 (columns 2 and 5) applies entropy balancing weights. Model 3 (columns 3 and 6) applies entropy balancing weights (EBW) and includes practice fixed effects (PCC FE). Standard errors (in brackets) are clustered by PCC. * *P<*0.05, ** *P<*0.01, *** *P<*0.001.

ED = emergency department. OOH = out-of-hours clinic.

^a^ *P<*0.05. ^b^ *P<*0.01*.* ^c^ *P<*0.001.

**Figure 1.**
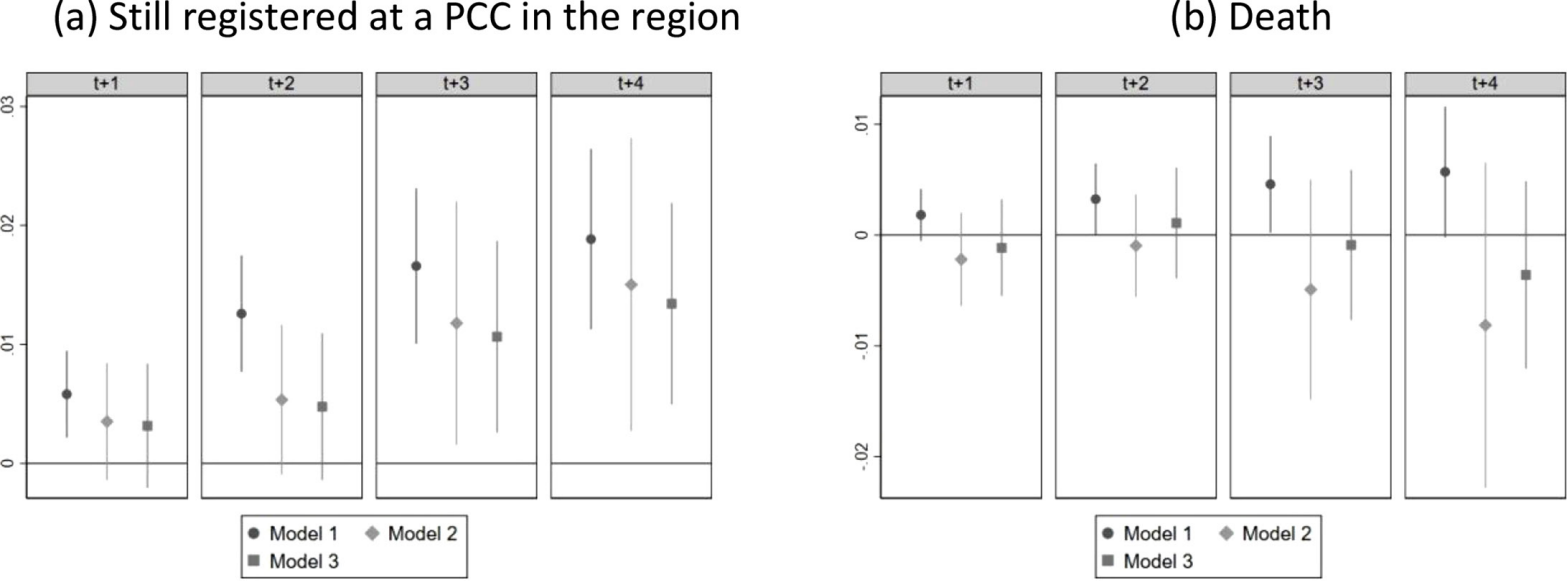
Differential attrition. *Note:* The figure shows estimates from model 1–3 contrasting patient with and without a named GP 1–4 years after the index date. Patients with an index date in 2015 lack data on mortality for t+4; otherwise, all models include the full study population. PCC = primary care centre.

There was no differential attrition in terms of mortality after adjusting for patient and PCC characteristics, but patients with a named GP were more likely to remain registered at a PCC in the region after 2 years (Figure 1). Notably, the sensitivity analysis using a shorter follow up did not indicate that patients with a named GP had higher UPC within 2 years; that is, during the period when the two groups were still equally likely to remain in the region. Furthermore, there was no statistically significant differential attrition, either in terms of death or discontinued registration in the region, for the subsample with at least three GP visits during the entire follow-up period (that is, the sample used in the analysis of the UPC).

## Discussion

### Summary

This study explored whether patients who were registered with a named GP at the onset of their first chronic condition had higher continuity of care at subsequent GP visits than patients without a named GP. Patients with a named GP had higher continuity of care, but the relationship vanished once the results were adjusted for patient and practice characteristics associated with the probability of having a named GP.

### Strengths and limitations

The key contribution of the study was to examine how continuity can be fostered for patients experiencing the onset of their first chronic disease. We were able to study the importance of a named GP thanks to the setting with group practices and variation in the prevalence of registration at a named GP. A strength was the access to detailed and longitudinal register data on all residents in the region, including practice registrations and diagnoses. We could identify patients who were registered with a named GP long before the onset of chronic disease, who were unlikely to have been assigned a named GP because of any previous health issues. The remaining imbalances in the study population were accounted for by the weighting and a within-practice approach. A limitation was that we did not study a mandated policy; practices and/or patients could influence the choice of registering patients at named GPs. Thus, if there would have been a strong correlation, it could have reflected that practices register specific patients with named GPs due to unobservable (to us) factors that correlate with their future need for continuity. However, the association was very weak. Possibly, the results reflect that PCCs who do not register patients with named GPs have other routines to ensure relational continuity. The results are unlikely to indicate that the scope for increasing continuity was exhausted, as patients only saw their usual provider on around half to two-thirds of all occasions. Another limitation was that we could not examine whether the UPC was the named GP, as different GP identifiers were used in the PCC enrolment register and the care utilisation data. However, making such a link was not crucial for the empirical analysis as specified.

A third limitation is that we could not explore the role of nurse continuity, which may be relevant for some of the diagnoses that were studied.

### Comparison with existing literature

Two evaluations of a reform which introduced the requirement to offer a named GP in the English NHS found no causal effect on the number of GP visits^21^ or continuity at visits.^16^ These studies had shorter follow-up periods (up to 2 years) than the present study. Furthermore, as most patients in the UK were already assigned to a named GP, the main change seen by the studied reform was that all patients, irrespective of their care need, were informed about who they were registered with.^16^ The current study focused on patients in a situation in which the presence of a named GP may be more likely to affect downstream care decisions: the period after the onset of a chronic disease. Yet, the authors found little to suggest that having a named GP helps build continuity.

Previous studies on survey data from Finland show that patients reporting that they have a named GP are more satisfied with their care^22^ and have greater access to care.^23^ With self-reported data, these analyses cannot separate the role of having a named GP from the role of continuity at visits, as patients may answer the survey based on their experience of both. The current study was able to isolate registration from continuity of care.

A Norwegian study reported a negative relationship between the duration of the patient–GP relationship and the risk of adverse events and out-of-hours visits.^10^ The association cannot be interpreted as a causal effect of the duration, as patients are not randomly assigned to durations of different lengths. This methodological insight is underlined by the present results, which indicate that registration with a named GP does not have a large impact per se on continuity of care or other outcomes.

### Implications for research and practice

In the current study setting, registration with a named GP was not mandated by the healthcare authority. The reasons why some patients were registered with a GP were therefore likely that i) it was the routine in their primary care practice, ii) it was requested by the patient (and accepted by the GP), or iii) a GP or other member of staff thought that the patient needed special attention, perhaps as a result of frailty. These models indicate that all these explanations may be relevant, and that the relationship between registration and continuity at visits is weak after accounting for these factors. This indicates that a policy that mandates that patients must be registered with named GPs is neither sufficient nor necessary to realise continuity in modern day primary care. To further establish causality, future research on the development of continuity of care after onset of a chronic disease should use quasi-experimental approaches.
